# The effect of educational intervention based on the PRECEDE-PROCEED model on self-care behaviors and quality of life of hypertensive patients

**DOI:** 10.3389/fpubh.2024.1410843

**Published:** 2024-07-18

**Authors:** Tayebeh Rakhshani, Zahra Tahmasebi, Leila Ghahremani, Amirhossein Kamyab, Ali Khani Jeihooni

**Affiliations:** ^1^Nutrition Research Center, Department of Public Health, School of Health, Shiraz University of Medical Sciences, Shiraz, Iran; ^2^Department of Public Health, School of Health, Shiraz University of Medical Sciences, Shiraz, Iran; ^3^Department of Health Education and Health Promotion, School of Health, Shiraz University of Medical Sciences, Shiraz, Iran; ^4^Faculty of Medicine, Fasa University of Medical Sciences, Fasa, Iran

**Keywords:** hypertension, educational intervention, self-care behavior, quality of life, PRECEDE-PROCEED model

## Abstract

**Background:**

To prevent the harmful consequences of hypertension and enhance the quality of life of hypertensive patients, the use of educational models is highly suggested. Therefore, the present study was designed to determine the effect of education based on the PRECEDE-PROCEED on self-care behaviors and the quality of life of hypertensive patients in Kazeroon city, Iran, in 2023.

**Methods:**

A total of 120 hypertensive individuals who were referred to Kazeroon city health centers participated in the current quasi-experimental study. The participants were divided into two experimental and control groups using a random sampling technique (60 participants in each group). The self-care behaviors questionnaire, the quality of life questionnaire, and a questionnaire based on the PRECEDE-PROCEED model were used as the data acquisition techniques. Both groups completed the questionnaires before and 2 months after the intervention. The educational program included a six-session, 50–60 min training program using three different teaching methods (speaking, Q&A, group discussion, and peer training) in health facilities. The data were examined using paired *t*, independent *t*, and chi-square statistical tests after being entered into the SPSS 24 statistical program.

**Results:**

Following the intervention, the experimental group showed significantly higher values in quality of life, knowledge, attitude, enabling and reinforcing factors, and self-care behaviors compared to the control group (*p* < 0.001 for all comparisons). The experimental group also exhibited a significant reduction in systolic blood pressure measures compared to the control group (*p* < 0.001).

**Conclusion:**

In the present study, education based on the PRECEDE-PROCEED model and focusing on blood pressure self-care behavior in patients with hypertension led to a decrease in their systolic blood pressure measures and improved their quality of life.

## Introduction

The high prevalence of hypertension around the world has made this disease a major health problem globally. Surveys conducted in Asia and the Pacific Ocean reveal that the prevalence of hypertension ranges from 5 to 47% in men and from 7 to 38% in women (1). Studies conducted in other countries have also reported this rate between 18 and 72% among women and men, respectively (2–5). Statistics reveal that in Iran, the prevalence of hypertension ranges from 8.4 to 49.5%, with middle-aged people having a rate of 22.06% and those over 55 having a rate of 49.5% (6). The prevalence of hypertension is 17.58% in the south of Iran, 1.4% in the northeast, 41.8% in the northern provinces, 18.9% in the central, and 24.5% in the southern regions (7–9).

One of the ways to control this disease is through the use of prescribed medication, and studies show that hypertension treatment can reduce diastolic blood pressure by 5–6 mm Hg. However, estimates suggest that approximately 50% of patients discontinue their treatment within a year (10). Therefore, adherence to treatment and self-care in hypertensive patients is crucial (11–13). The evidence indicates the impact of patients, service providers, and service delivery systems on the self-care behaviors of hypertensive patients. In order to prevent the harmful consequences of hypertension, it is vital to pay attention to the development of necessary education about the nature of hypertension, the seriousness of its continuous follow-up and control, as well as self-care (14). In addition to the complications of hypertension, it also affects the patient’s quality of life and performance and sometimes even leads to disability (15). This continuous chain causes a lack of independence in performing daily tasks and leads the affected person to depend on others. In addition to the direct effects of inactivity, a person’s inactivity can also lead to diseases such as cardiovascular disease, obesity, diabetes, and osteoporosis. The destructive effects of the disease on physical disability and quality of life are considerable (16).

Recent decades have placed significant emphasis on the quality of life and its impact on health. This concept encompasses various aspects of life, including physical, emotional, cognitive, and social aspects, and refers to an individual’s level of satisfaction with their life facilities (17). Quality of life is the set of conditions that make it possible to live well in such a way that a person is able to perform their daily activities in a suitable physical, mental, and social condition, and the patient is also satisfied with the effectiveness of treatment, disease control, or rehabilitation. Quality of life encompasses all life functions, such as emotional, physical, chemical, pain, fatigue, and so on (18).

Along with diseases like hypertension, self-care emphasizes the maintenance of human health in three areas: physical, mental, and social. It refers to the individual activities people perform to maintain their overall health and well-being (19). Despite the need for physicians to take measures to control these patients and improve their quality of life, treatment with medication does not have much effect on the quality of life of these patients (20). Encouraging self-care measures in these patients and planning based on potential ability is of particular importance (20). Several interventions have been made to improve self-care; however, self-care in hypertension is complicated, has low effectiveness, and is expensive and time-consuming. This type of intervention is also hard to determine which part worked best (21). Among the many models of health education, the PRECEDE-PROCEED model is an effective one (21).

The PRECEDE-PROCEED model is widely recognized for its application in designing, implementing, and evaluating health education and promotion interventions. It has been effectively utilized in various settings, including community health programs and management of chronic diseases (22). Originally, this model was applied to training programs for diverse populations, including patients in clinical settings and employees within corporate wellness programs. For instance, studies have shown its efficacy in workplace wellness initiatives aimed at improving employee health outcomes (23, 24). Its adaptability to different contexts allows for its application in clinical settings like ours. Therefore, we adapted this model specifically to focus on hypertensive patients, tailoring the intervention to address the unique predisposing, enabling, and reinforcing factors that influence self-care behaviors and quality of life in these patients. This adaptation involves customizing educational materials and activities to meet the needs of hypertensive patients, ensuring relevance and effectiveness in promoting blood pressure control and their overall well-being.

In addition to selecting an educational model, prioritizing a peer-oriented approach is also necessary, as this approach can foster effective communication among peers, encouraging them to adhere to treatment and dietary guidelines (25). On the other hand, peer-oriented education on health issues creates a supportive environment and has long-term effects on the self-management of chronic diseases. Therefore, adopting a peer-oriented model can improve the management of hypertensive patients and reduce the costs of the disease and its complications (25). Some studies have highlighted the effectiveness of interventions in controlling hypertension. For instance, studies by Hou et al. (26), Kim et al. (27), and Jafar et al. (28) indicated that health interventions can effectively control hypertension (26–28). All in all, the need for a program based on an educational model to guide patients correctly to the point of evaluation is undeniable. Therefore, according to the results of the studies, the purpose of the study was to increase self-care behaviors in order to increase the quality of life of patients with hypertension who were referred to the health centers in Kazeroon City, Iran, in 2023.

## Methods

### Research design

This quasi-experimental study was executed on 120 hypertensive patients in Kazeroon city, Iran, in 2023. Their disease was previously verified by a physician. The patients were visited by health care staff each month and every 3 months by a physician. The criteria for entering the study were patients diagnosed with primary hypertension who had at least a 6-month history of hypertension, referring to comprehensive health centers, having appropriate visual and auditory performance, and willingness to participate in the study. The exclusion criteria included unwillingness to cooperate at any time during the study and failure to participate in at least two training sessions.

### Sample size and sampling method

The average comparison formula in two communities and the findings of Babaei-Sis et al.’s (2016) study were used to calculate the number of participants. The experimental group’s mean and standard deviation for self-care behaviors were 30.5 ± 2.8, while the control group’s were 29.27 ± 3.3. For every group of 60 participants, a 10% attrition rate, 80% test power, and a 95% confidence level were set as requirements in order to compute the sample size (29).


$$n=\frac{{\left(Z_{1-\frac{\alpha }{2}}+Z_{1-\beta }\right)}^{2}\left(\delta_{1}^{2}+\delta_{2}^{2}\right)}{{\left(\mu_{1}-\mu_{2}\right)}^{2}}$$

Z_1 − α/2_: Z value corresponding to the desired confidence level (1.96).

Z_β_: Z value corresponding to the desired power (0.84).

δ_1_: The standard deviation of the experimental group (2.8).

δ_2_: The standard deviation of the control group (3.3).

μ_1_: The mean of the experimental group, (30.5).

μ_2_: The mean of the control group, (29.27).

The cluster random sampling method was used for this study. From the total number of health houses in Kazeroon city, we randomly selected four health centers, two for the experimental group and two as controls. Next, we randomly selected the desired samples from these health centers.

### Instruments

In order to collect information, a questionnaire containing personal information about people, a questionnaire made by the researcher based on the PRECEDE-PROCEED model, and the quality of life questionnaire were used.

### Questionnaire of demographic variables

Demographic characteristics included age, sex, marital status, education, occupation, family history of hypertension, and systolic and diastolic blood pressures before and after the intervention. A sphygmomanometer (mercury sphygmomanometer) was used to measure the blood pressures of the participants. Blood pressure was measured by heath care staff. Blood pressure was measured from the right hand in a sitting position; the person’s hand was not bent, fisted, or hanging; and the client’s legs were supported (the person was sitting comfortably, leaning his back, and the soles of his feet were on the floor).

### Questionnaire based on the PRECEDE-PROCEED model

This questionnaire contains knowledge (10 yes/no questions such as definition of hypertension, symptoms of hypertension, and complications of hypertension), attitude (10 questions such as complications of hypertension, use of hypertension drugs, lifestyle in hypertension patients), reinforcing factors (10 questions about access to information about self-care behaviors, access to medications), and enabling factors (10 questions such as: Do you have enough financial resources to provide your medications? Is access to information about the self-care behaviors of the disease easy for you?) and self-care behaviors (15 questions such as, Do you regularly participate in physical activity, for example, 4–5 walks for 30 min each time? Do you read food labels about their salt content?) These questions were scored on a Likert scale ranging from completely disagreeing with a score of 1 to completely agreeing with a score of 5. The range of scores for knowledge was 10–20, attitude was 10–50, reinforcing factors were 10–50, enabling factors were 10–50, and self-care behavior was 15–75.

### Determining the validity and reliability of the researcher-made questionnaire

The questionnaire’s validity was assessed using both quantitative and qualitative methods. Twelve experts (outside the research team) in the fields of health education and promotion were given the questionnaire for the qualitative face validity component. These experts included two experts, one physician, and one epidemiologist. Two coefficients of content validity ratio and content validity index were employed to verify the validity quantitatively for the part of validity that was quantitative.

The current study employed internal consistency methodologies to assess the tool’s reliability. The questionnaire was given to 30 participants who met the study’s eligibility requirements in order to ascertain the internal correlation of the tool’s various components. The results of this analysis were obtained using SPSS version 24, and the Cronbach’s alpha coefficient was calculated for both the questionnaire’s total content and the PRECEDE-PROCEED model’s constructs. The reinforcing factors had a Cronbach’s alpha of 0.81, the enabling factors 0.83, the self-care behavior 0.76, and the questionnaire as a whole had a 0.84.

### Quality of life questionnaire

The 12-question version is a condensed version of the 36-question quality of life questionnaire, which is frequently used in research. Warr, Kasinski, and Keller created the 12-question version of the quality of life questionnaire back in 1996. There are eight subscales in this survey. Because the number of components is limited, the individual’s total scores are frequently used. The current survey looks at a person’s overall health, physical function, emotional difficulties, physical discomfort, social function, vitality and vital energy, and mental health in order to assess their quality of life. In Arabi et al.’s study, the questionnaire had a Cronbach’s alpha of 95%, indicating the validity and reliability of the Persian version of the questionnaire measuring the quality of life of impaired individuals (30).

### Procedure

Before designing the training program, all participants completed a questionnaire on blood pressure self-care behavior. Finally, in order to improve the experimental group’s hypertension self-care behavior, the training, which lasted six sessions for 50 to 60 min each, was structured around three different teaching modalities in health centers: lecture, group discussion and Q&A, and peer education. The data was then completed once more and compared with the pre-intervention data 2 months following the intervention.

A team of experts, including a dietitian, a psychologist, and a physician, carried out the courses. Each member of the team focused on developing materials for instruction related to improving self-care practices for hypertension. The trainings included group discussions, illustrations, Q&As, and a training film that was created based on the Iran Ministry of Health training manual. Meetings were open to the patient’s relatives, who have authority over them. Furthermore, during the fourth and fifth sessions, the participants were requested to share their experiences with hypertension and their self-care practices in a peer-focused manner (Table 1).

**Table 1:** The educational intervention program.

Session	Goal	Subject	Length (minutes)	Strategy	Tutor
1	Improving the level of knowledge and attitude of patients	Learning about each other and the psychologist from the group members aims stated being aware of hypertension, how it affects patients’ sensitivity, and how to assess each patient’s blood pressure disease severity	60	Lecture, Q&A	Experts and researcher
2	Introducing the PRECEDE-PROCEED model	Introducing enabling variables, recognizing barriers, and enhancing enabling factors on an individual basis	60	Lecture, group discussion, Q&A	Psychologist and researcher
3	Education based on the model	Overview of the advantages and challenges of blood pressure management	60	Educational film and poster	Consultant expert and researcher
4	Peer-based education	Expression of self-care practices acquired from books, the internet, and all other educational resources	60	Group discussion	Patients
5	Instructing the quality of life	What quality of life is, how it relates to hypertension, and what factors influence it	60	Group discussion	Patients
6	Sum up and review	Going over the earlier content, summarizing it, and making a final assessment	60		Researcher

### Data analysis

SPSS version 24 was used to analyze the data. The Kolmogorov–Smirnov test was originally used to determine whether the data were normal. The data were described using frequency indices, mean, and standard deviation. The mean values in the two groups’ pre- and post-interaction were compared using independent *t*-tests, chi-square tests, and paired *t*-tests (*p* < 0.05).

## Results

According to the Kolmogorov–Smirnov test, the normality of the data was not rejected for every variable. Table 2 displays the demographic data for the two groups in the study. The mean age of participants was 50.95 ± 10.09 for the experimental group and 51.88 ± 11.44 for the control group (*p* = 0.648). A chi-square test revealed no substantial variation in gender (*p* = 0.131), marital status (*p* = 0.426), education (*p* = 0.215), occupation (*p* = 0.451), or familial history of hypertension (*p* = 0.261) between the experimental and control groups. Age differences between the experimental and control groups were not statistically significant (*p* = 0.648), corresponding to the t-test (Table 2).

**Table 2:** Comparison of the frequency distribution of the primary variables of the participants in the study.

Variable		Experimental group	Control group	*p*-value
		Frequency (%)	Frequency (%)	
Sex	Female	39 (65)	42 (70)	0.131^*^
	Male	21 (35)	18 (30)	
Marital status	Married	54 (90)	51 (85)	0.426^*^
	Single or widowed	6 (10)	9 (15)	
Education	Elementary school	28 (46.66)	34 (56.66)	0.215*
	Secondary school	26 (43.33)	16 (26.66)	
	University degree	6 (10)	10 (16.66)	
Occupation	Employee	15 (25)	12 (20)	0.451^*^
	Housewife	35 (58.33)	38 (63.33)	
	Self-employed	10 (16.66)	10 (16.66)	
Family history of hypertension	No	42 (70)	39 (65)	0.261^*^
	Yes	18 (30)	21 (35)	

* Chi-square test.

Table 3 displays the means and standard deviations of the constructs of the PRECEDE-PROCEED model in the two groups. The independent t-test showed that there was no significant difference between the two groups in terms of knowledge (*p* = 0.125), attitude (*p* = 0.216), enabling factors (*p* = 0.325), reinforcing factors (0.145; *p* = 0.687), or self-care behavior (*p* = 0.687) before the intervention. However, following the training, there was a substantial disparity among the two groups’ knowledge, attitude, enabling factors, reinforcing factors, and self-care behaviors (*p* = 0.001; Table 3).

**Table 3:** Comparison of the mean and standard deviation of the constructs of the PRECEDE-PROCEED model pre- and post-intervention.

Constructs	Group	M ± SD	M ± SD	*p*-value^*^
		Before intervention	Two months after intervention	
Knowledge	Experimental	12.15 ± 2.41	16.21 ± 3.22	0.001
	Control	12.26 ± 3.41	12.84 ± 3.12	0.926
	*p*-value^**^	0.125	0.001	
Attitude	Experimental	26.81 ± 6.71	34.51 ± 3.25	0.001
	Control	25.81 ± 6.84	25.51 ± 6.25	0.421
	*p*-value^**^	0.216	0.001	
Enabling factors	Experimental	27.03 ± 5.25	37.19 ± 4.18	0.001
	Control	26.03 ± 4.01	26.19 ± 3.98	0.116
	*p*-value^**^	0.325	0.001	
Reinforcing factors	Experimental	31.65 ± 7.61	36.16 ± 4.12	0.001
	Control	32.07 ± 6.15	31.88 ± 4.04	0.226
	*p*-value^**^	0.145	0.001	
Self-care behaviors	Experimental	28.12 ± 4.29	38.25 ± 5.61	0.001
	Control	28.94 ± 3.12	28.16 ± 4.13	0.425
	*p*-value^**^	0.687	0.001	

* Paired *t* test.

** Independent *t*-test.

Table 4 displays the mean and standard deviation of the two study groups’ systolic and diastolic blood pressures. The independent t-test results showed a significant difference (*p* = 0.001) in the systolic blood pressure measures between the two groups after the intervention, while there was no difference among the two groups before the intervention (*p* = 0.731).

**Table 4:** Comparison of the mean and standard deviation of systolic and diastolic blood pressure measures pre- and post-intervention.

Constructs	Group	M ± SD	M ± SD	*p*-value^*^
		Before intervention	Two months after intervention	
Systolic blood pressure	Experimental	141.39 ± 9.26	128.50 ± 11.17	0.001
	Control	143.21 ± 8.72	141.42 ± 16.25	0.218
	*p*-value^**^	0.731	0.001	
Diastolic blood pressure	Experimental	84.50 ± 5.71	83.52 ± 4.15	0.284
	Control	85.35 ± 5.71	84.50 ± 3.16	0.405
	*p*-value^**^	0.265	0.148	

* Paired *t* test.

** Independent *t*-test.

Table 5 displays the mean and standard deviation of the quality of life for each group. Before the intervention, the scores did not differ substantially among the two groups (*p* = 0.226), but a notable change was observed after the intervention (*p* = 0.001). These findings were based on the results of the independent t-test.

**Table 5:** Comparison of the mean and standard deviation of the quality of life pre- and post-intervention among the two groups.

Constructs	Group	M ± SD	M ± SD	*p*-value^*^
		Before intervention	Two months after intervention	
Quality of life	Experimental	31.82 ± 3.60	36.84 ± 3.25	0.001
	Control	31.53 ± 3.40	31.51 ± 3.25	0.369
	*p*-value^**^	0.226	0.001	

* Paired *t* test.

** Independent *t*-test.

## Discussion

This study was designed to investigate the effect of education based on the PRECEDE-PROCEED model on the self-care behaviors of hypertension in a group of hypertensive patients. In this study, after the intervention, a significant increase in knowledge was found in the experimental group compared to the control group. The PRECEDE-PROCEED model encompasses efforts to understand the variables influencing behavior and related processes in health-related fields. This model is considered a valuable tool in the research process. This pattern increases a person’s sensitivity to disease, leading to adherence to self-care behaviors. This finding is consistent with the results of the studies by Yuting et al., Jullmusi et al., Ignasimuthu et al., and Adawiyah et al. (31–34).

Following the intervention, the experimental group’s attitude sharply improved compared to the control group. In general, the PRECEDE-PROCEED model, which is a cognitive model that evaluates the way people respond to health-threatening factors, leads to an increase in the intensity of a perception. Our findings are consistent with the results of studies by Islam et al. examining the relationship between physical activity and attitude towards lowering blood pressure, Zahed et al.’s study examining the effect of using mobile-based interventions on increasing the attitude of hypertensive patients, and Rodrigues et al. (35–37).

In this investigation, the experimental group experienced a noticeable rise in reinforcing factors following the intervention, in contrast to the control group. One of the structures of the PRECEDE-PROCEED model is the recognition of reinforcing factors, and during the training, the patients gained detailed knowledge of reinforcing factors, achieved self-care of their blood pressure, and therefore may try to fix it. This finding is in agreement with the results of Hauspurg et al., Jiang et al., and the intervention of Englert et al., with the aim of reducing blood pressure (38–40).

Following the intervention, there was a noticeable increase in the experimental group’s enabling factors compared to the control group. Patients have learned in the training sessions that they can lower the costs associated with their disease by adhering to a few simple training guidelines (such as taking their medications as prescribed or complying with their treatment plans). With this method, they might reduce their disease’s complications, which would eventually increase the benefits of their self-care practices. This result is consistent with the findings of the Avegno et al. research, Kappes et al., and the Sampin et al. study (41–43).

The experimental group showed a significant increase in self-care behaviors after the intervention compared to the control group. It seems that the training designed based on the PRECEDE-PROCEED model has increased the performance and health beliefs of hypertensive patients in the field of self-care behavior. This result is similar to what Sampain et al., Sanya et al., and Gupta et al., who wanted to see how diet-based interventions could help hypertensive patients learn more, and Koleva et al. found (43–46).

According to the results, the intervention led to a decrease in the average systolic blood pressure in the experimental group compared to the control group. In justifying this finding, it can be stated that self-care leads to lifestyle improvement, and lifestyle improvement as a causal relationship leads to a significant reduction in systolic blood pressure in patients with hypertension, so performing self-care behaviors and teaching them based on the PRECEDE-PROCEED model can be added to people’s drug treatment as the first line of treatment in patients with hypertension. This finding is consistent with the study of Khani Jeihooni et al., the study of Bulto et al., and the study of Thapa et al. (47–49).

Although non-significant, the experimental group’s average diastolic blood pressure decreased as a result of the intervention in this study. Health interventions that center on self-care behavior generally result in patient compliance and a greater desire to follow his diet; additionally, the patient’s diastolic blood pressure lowers, and this training increases the patient’s compliance with treatment. Similarly, in studies by Falah et al., He et al., and Cartwright et al., the diastolic blood pressure measures decreased notably as a result of their intervention (50–52).

In the current study, the experimental group’s quality of life improved compared to the control group as a result of the intervention. Training based on the PRECEDE-PROCEED model is responsible for the increase in the experimental group’s average behavior scores when compared to the control. Since lifestyle modification plays a substantial role in quality of life, education centered on PRECEDE-PROCEED will ultimately result in adjusting the lifestyle of hypertensive patients and lowering their blood pressure, which can lead to an improvement in their health conditions. This finding is in line with the results of the study by Weng et al., with the aim of educational intervention on quality of life (53).

### Strengths and weaknesses

This study’s strengths include the way in which health centers and patients, as well as the peer group, were involved in its implementation; it also has the advantage of designing educational interventions based on pre-test results and utilizing a variety of instructional techniques during the educational sessions. However, the study’s short-term follow-up on the program’s effects could serve as a weakness.

## Conclusion

In the present study, education based on the PRECEDE-PROCEED model and focusing on blood pressure self-care behavior in patients with hypertension led to a decrease in their systolic blood pressure measures and improved their quality of life. Given the importance of health education in promoting blood pressure control and preventing hypertension complications, and the importance of monitoring blood pressure-related behaviors in preventing these complications, prioritizing education in health fields is highly suggested. This includes education in a broader sense and the use of a variety of educational resources. Therefore, it is suggested that longer interventions be carried out in hypertensive patients.

## Data availability statement

The data that support the findings of this study are available from the corresponding author upon reasonable request.

## Ethics statement

The studies involving humans were approved by Shiraz university of medical sciences. The studies were conducted in accordance with the local legislation and institutional requirements. The participants provided their written informed consent to participate in this study.

## Author contributions

TR: Writing – original draft, Visualization, Project administration, Methodology, Conceptualization. ZT: Writing – original draft, Supervision, Software, Resources, Project administration, Methodology, Investigation, Formal analysis, Data curation, Conceptualization. LG: Writing – review & editing, Visualization, Project administration, Methodology, Conceptualization. AmK: Writing – review & editing, Software, Resources, Methodology, Investigation, Formal analysis, Data curation, Conceptualization. AlK: Writing – review & editing, Writing – original draft, Visualization, Validation, Supervision, Software, Resources, Project administration, Methodology, Investigation, Formal analysis, Data curation, Conceptualization.
